# P04.79. Navigating breast and cervical cancer screening services for Tongan women in Southern California

**DOI:** 10.1186/1472-6882-12-S1-P349

**Published:** 2012-06-12

**Authors:** R Peters, L Beck, L Fifita, B Hui, V Tui`one, C Page, R Sur, S Tanjasiri

**Affiliations:** 1 California State University Fullerton, Fullerton, USA; 2 Tongan Community Service Center/Special Service for Groups, Hawthorne, USA

## Purpose

Patient Navigation (PN) has the potential to increase timely utilization of cancer prevention, early detection and treatment services among underserved populations. Few such navigators exist in community-based settings. Tongans in Southern California experience some of the largest cancer health disparities in the state. In addressing breast and cervical cancer, a pilot project was launched with the aim to increase cancer screening, treatment and support for Tongan American women ages 40 and above through cancer-related PN services.

## Methods

The project consisted of assessment of patient-level barriers to screening, identifying and recruiting women for screening, and providing screening support and PN services to patients and their families. A mixed methods approach was used to analyze secondary data collected from Patient Intake and Tracking Forms. These forms collected data on demographics, health history, current cancer screenings and navigation services provided.

## Results

A culturally and linguistically tailored breast and cervical cancer education and navigation project was established. Data showed major barriers to screening; 39.4% were uninsured, 35.1% had a high school diploma or less, 29.8% were non-English speaking, and 27.7% were undocumented. Results found that (1) Pap tests increased from 26.2% to 53.2% at the end of the project; (2) mammogram screenings increased from 29.1% to 62.0%; (3) scheduling and coordination of screening appointments was the most frequent service (34%), followed by reminders of upcoming screening appointments (18.1%); and (4) the average number of contacts per patient was 2.36 suggesting multiple contacts are needed to overcome barriers to screening.

## Conclusion

The pilot demonstrates screening needs of this underserved population, confirms lack of access as a primary barrier to screening, highlights the importance of PN in supporting women’s screening, and underscores the importance of policies that support patient navigation in conjunction with the Patient Protection and Affordable Care Act, which expands health access to the medically underserved.

